# Gut and Brain: Investigating Physiological and Pathological Interactions Between Microbiota and Brain to Gain New Therapeutic Avenues for Brain Diseases

**DOI:** 10.3389/fnins.2021.753915

**Published:** 2021-10-12

**Authors:** Gabriele Deidda, Manuele Biazzo

**Affiliations:** ^1^The BioArte Limited, Life Sciences Park, San Gwann, Malta; ^2^Department of Physiology and Biochemistry, Faculty of Medicine and Surgery, University of Malta, Msida, Malta; ^3^SienabioACTIVE, University of Siena, Siena, Italy

**Keywords:** brain disorders, neurodevelopmental disorders, neurodegenerative diseases, microbiota, gut-brain, gut dysbiosis, prebiotics, probiotics

## Abstract

Brain physiological functions or pathological dysfunctions do surely depend on the activity of both neuronal and non-neuronal populations. Nevertheless, over the last decades, compelling and fast accumulating evidence showed that the brain is not alone. Indeed, the so-called “gut brain,” composed of the microbial populations living in the gut, forms a symbiotic superorganism weighing as the human brain and strongly communicating with the latter *via* the gut–brain axis. The gut brain does exert a control on brain (dys)functions and it will eventually become a promising valuable therapeutic target for a number of brain pathologies. In the present review, we will first describe the role of gut microbiota in normal brain physiology from neurodevelopment till adulthood, and thereafter we will discuss evidence from the literature showing how gut microbiota alterations are a signature in a number of brain pathologies ranging from neurodevelopmental to neurodegenerative disorders, and how pre/probiotic supplement interventions aimed to correct the altered dysbiosis in pathological conditions may represent a valuable future therapeutic strategy.

## Introduction

The brain is the organ generating and controlling any behavior of an individual. It is composed of billions of different neuronal and non-neuronal cell populations interconnected by extremely complex structural networks. The activity of these networks generates a plethora of lower and higher brain functions, ranging from simple motor reflexes in invertebrates and complex motor actions in non-human primates to sensory perceptions and higher cognitive functions in humans (for example, attention and decision-making). Importantly, if a normal (healthy) structural and functional brain is surely the means to achieve normal brain functions in normal physiological conditions, it is also clearly true that defects causing structural and/or functional alterations in the brain are responsible for brain dysfunctions in pathological conditions. Given the extreme complexity of the human brain, it does not come as a surprise that most of the human pathologies described so far are related to the brain.

**FIGURE 1:**
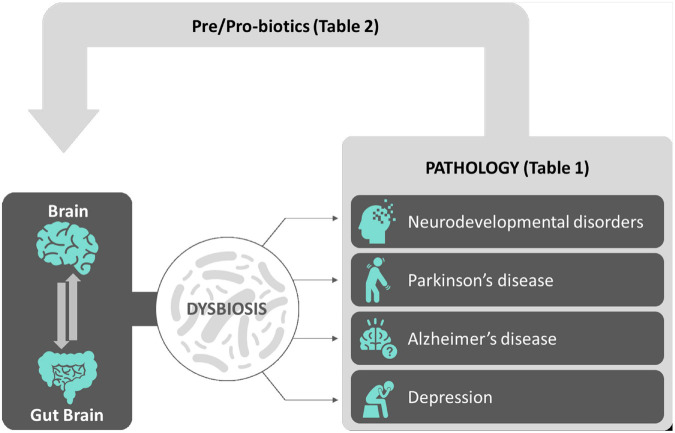
Sketch depicting an overview of the review. The complex interactions between the brain and the gut–brain (gut microbiota) contribute to the physiological balance achieved during normal brain functions. Alterations of gut microbiota microflora leading to gut dysbiosis have been correlated to a number of brain conditions including neurodevelopmental disorders, neurodegenerative diseases, and depression, described both in animal models and in mankind (see Table 1 for a summary). Among possible effective new therapeutic avenues, pre/probiotic supplements aimed to correct gut microflora dysbiosis and to reinstate neurophysiological balance in neuropathological background had been shown so far to be effective in a number of clinical trials (see Table 2 for a summary).

Notably, the brain is not alone. Indeed, from the first pioneering studies on the microbial ecology back to more than three decades ago (Savage, 1977), the following extensive scientific research in the domain of the so-called *microbiome* (or biome) shed light on and revealed the essential contribution of microbial communities populating and living within the human body into the general physiological functions and health of an individual (Liang et al., 2018). Over 98% of our body’s microbes are located within the gastrointestinal (GI) tract. The microorganisms specifically living and residing in the gut, referred to as *gut microbiota*, represent a dynamic population of microbes forming a symbiotic superorganism (approximately 10^14^ cells) containing 100 times the number of genes of the human genome and weighing approximately the same as the human brain. The gut microbiota communicates with the brain *via* the gut–brain axis. The *gut brain* has recently become a major player both in regulating normal brain functions in normal physiological conditions and in contributing in being a risk factor/causative for neuropathological conditions (Ma et al., 2019). In this review, after a general outline of the microbiome profiles in the brain in physiological (health) and pathological conditions (disease) spanning the whole ontogeny (from development to adulthood), we will propose how pre/probiotics supplementation to people with neurological disorders will eventually provide in the near future a new therapeutic tool aiming to either decrease the probability that a healthy brain will shift into pathology or to slow down an existing neuropathology (see Figure 1 for an overview).

## Microbiota and Gut Brain

Our world is home to a plethora of microbial species ranging from archaea to viruses, from fungi to parasites (protozoa and worms) that form complex microbial communities called *microbiota*. These communities interact with each other, cross act within an ecological niche, and form a complex of interconnected networks that communicate, cross-feed, recombine, co-evolve, and cooperate not only with each other, but also with the animal species that host them (Layeghifard et al., 2017). As humans, we are not an exception; in fact, our body is also home to communities of microorganisms formed by thousands of microbial species. For adaptive and host co-evolution strategies, the microbial community of each area of the human body is unique in its composition and it presents different microbial ecology niches (Lloyd-Price et al., 2016). These microbial communities can be beneficial to the human host (Mao et al., 2013; Knights et al., 2014) and have indubitably implications in both health and disease (Maynard et al., 2012). Already from the early infant life, the microbial colonization of the infant gut (who inherits the own microbiota from the mother at birth) is essential for the early postnatal development of the innate immune system (Arboleya et al., 2012; Gomez de Aguero et al., 2016). In fact, the intestinal microbiota profile of preterm infants showed higher levels of facultative anaerobic microorganisms and reduced levels of strict anaerobes such as *Bifidobacterium*, *Bacteroides*, and *Atopobium* compared with that of full-term, vaginally delivered, breast-fed infants. Later in life, these microbial communities can significantly affect human health and even modulate the clinical outcomes of infections (Liang et al., 2018). *Vice versa*, alteration/perturbations/imbalance in the microbial community composition (*dysbiosis*) can lead to unfavorable responses and/or pathological conditions in the host, including modifications in relevant behaviors. After birth, the microbial flora changes depending on the dietary habits and environment effects (Koh et al., 2016; Wahlstrom et al., 2016).

The *human gut* is home to a variety of bacteria, archaea, fungi (mostly yeasts), microbial eukaryotes (usually *Blastocystis* and a variety of pathogenic and non-pathogenic taxa), and viruses/phages. This collection of microbes is called the *gut microbiota*, and their genes are complexly called the *microbiome* (Turnbaugh et al., 2007). The general composition of gut microbial community included the following five phyla: *Bacteroidetes*, *Firmicutes*, *Actinobacteria*, *Proteobacteria*, and *Verrucomicrobia* (The Human Microbiome Project Consortium, 2012). In the healthy gut, the anaerobic *Bacteroidetes* and *Firmicutes* contribute to more than 90% of the total bacterial species, and their ratio changes across different individuals mostly because of (i) differences in individual (host) genomes and (ii) environmental factors (antibiotic use, lifestyle, hygiene, and diet) (The Human Microbiome Project Consortium, 2012). Dysbiosis of the human gut microbiome is associated with a wide range of pathologies, including obesity (Perry et al., 2016), diabetes (Gulden et al., 2015), diarrhea (Pop et al., 2014), and irritable bowel syndrome (IBS) (Jalanka-Tuovinen et al., 2014). The concept of microbial dysbiosis also includes the microbiome bacteriophage components that are implicated in a wide range of physiological (health) and pathological conditions (Manrique et al., 2017). Bacteriophages, also called phages, parasite their host, the bacteria, and do play an important regulator role in the host–microbiome interactions through horizontal gene transfer and antagonistic coevolution (Scanlan, 2017; Duerkop, 2018).

Nevertheless, the gut microbiota is not an isolated community that simply lives in the gut and within the host, but it does profoundly communicate with the other organs (even distant ones) by means of microbial signals transmitted across the intestinal epithelium and *via* different pathways, for example, (i) the trimethylamine (TMA)/trimethylamine N-oxide (TMAO), (ii) the short chain fatty acids (SCFAs), and (iii) the primary and secondary bile acid (BAs) pathways (Pluznick et al., 2013). Gut–brain communication occurs also *via* the vagus nerve. Microbiota-derived molecules act either by functionally interacting with other endocrine hormones (i.e., ghrelin, leptin, glucagon-like peptide 1, and peptide YY) or by stimulating the parasympathetic nervous system, thereby affecting glucose homeostasis and other metabolic processes linked to the production of microbiota-generated metabolites promoting metabolic benefits, as, for example, in promoting body weight and glucose control (demonstrated in animal models) (De Vadder et al., 2014). Despite the presence of the brain–blood barrier (BBB), these molecules can reach the brain thanks to the brain–gut–microbiota axis: a bidirectional communication system enabling gut microbes to communicate with the brain and the brain with the gut, and it can have a profound effect on brain physiological state (Rhee et al., 2009). The mechanisms of signal transmission are complex and include neural, endocrine, immune, and metabolic pathways (Grenham et al., 2011; El Aidy et al., 2015). This ability of the microbes to influence brain-related behaviors suggests that they induce the release of host immune factors (immune-mediated, i.e., cytokines and inflammatory mediators) targeting both the central nervous system (CNS) and the enteric nervous system (ENS) (Wood, 2004). Moreover, bacteria can themselves synthetize and regulate the level of many neurotransmitters in the brain including major (GABA, glutamate) and neuromodulatory ones (serotonin, dopamine, and norepinephrine) that are known to play pivotal roles in social behavior (Liu et al., 2017; Strandwitz et al., 2019). For example, most of the serotonin in the body is produced in the gut by enterochromaffin cells under the influence of the microbiome (Yano et al., 2015), and *Bifidobacterium infantis* has been shown in animal models to elevate the plasma levels of tryptophan (the precursor of the serotonin synthesis), thus influencing those brain systems involved in mood disorders (Desbonnet et al., 2010). In this review, we will specifically focus on the relation between the gut microbiota and the brain.

## Gut Brain and Brain Pathologies

The ability of pathogens to profoundly influence and affect relevant brain behaviors of their hosts has been described a long time ago in the literature. In rodents, for example, *Toxoplasma gondii* parasitic infection ultimately results in a dramatic (and fatal) decrease in anxiety-like behavior in the infected rodents that no longer show fear of feline predators (Berdoy et al., 2000). Not only (external) pathogens that cause infections and alter animal behavior, but also microbial species residing within the gut can have profound effects, revealing the recently engendered use of the term “mind-altering bugs” that in fact stresses how variations and changes in the composition of the gut microbiota can influence normal brain physiology and behavior, and also contribute to diseases ranging from inflammation to obesity to the regulation of anxiety, mood, cognition, and pain (Cryan and Dinan, 2012). For instance, the gut microbiota can influence social interactions by acting on the nutritional behavior of individual animals (Pasquaretta et al., 2018). Low oral doses of *Campylobacter jejuni*, a bacterium within the gut, induce anxiety-like behavior in mice through a vagal-mediated pathway in the absence of any immune activation (Goehler et al., 2005). In humans, people with inflammatory bowel diseases (characterized by altered microbial diversity) show alteration in anxiety and depression behaviors (Blanchard et al., 1990).

Moreover, the host microbiota constantly control maturation and function of macrophages in the brain (Erny et al., 2015), the microglia cells critically involved in brain diseases.

The gut microbiota serves thereby as a proper “microbial organ” (i) being in continuous communication with the neurophysiological system of the host and (ii) acting as a driver of homeostasis and disease (Lyte, 2010). Thus, if an unhealthy gut might play a pivotal role in an unhealthy brain and contribute to neurodegenerative diseases (Spielman et al., 2018), consequently the modulation of the gut microbiota may be a tractable strategy for the development of new therapeutic strategies for complex neurological disorders (Cryan and Dinan, 2012). Table 1 summarizes gut microbiota alterations in animal models and in mankind across brain disorder and diseases.

**TABLE 1:** Summary of gut microbiota alterations in animal models and in mankind across brain disorders and diseases.

Brain disorder/disease		Gut dysfunctions and microbiota alterations	Selected references
Autism spectrum disorders	*Animal models*	The maternal high-fat diet protocol in mice results in social defects (autistic-like) in the offspring depending on *Lactobacillus reuteri* dysbiosis.	Buffington et al., 2016
	*Mankind*	Abdominal pain, constipation, vomiting.	Molloy and Manning-Courtney, 2003
		↑ *Desulfovibrio* species ↑ *Bacteroides vulgatus* ↑ SCFA ↑ *Clostridium bolteae*	Finegold et al., 2010; Wang et al., 2012; Cao et al., 2013; Pequegnat et al., 2013
Schizophrenia spectrum disorders	*Mankind*	↑ *Lactobacillus* that positively correlates with the severity of psychotic symptoms ↑ *Saccharophagus*, *Ochrobactrum*, *Tropheryma*, *Halothiobacillus*, *Deferribacte*r, and *Halorubrum* (at the genus level) ↑ *Bifidobacterium* and *Ascomycota* ↑ *Candida albicans* (in males only) ↓ *Anabaena*, *Nitrosospira*, and *Gallionella* (at the genus level)	Castro-Nallar et al., 2015; Severance et al., 2016; Schwarz et al., 2018
Rett syndrome	*Mankind*	Nutritional problems, pain, constipation, and chewing difficulties.	Motil et al., 2012
		↑ *Bifidobacterium*, *Clostridia* (at the taxa level), including *Anaerostipes, Clostridium XIVa*, and *Clostridium XIVb* ↑ *Erysipelotrichaceae*, *Actinomyces*, *Lactobacillus*, *Enterococcus*, *Eggerthella*, and *Escherichi*a/*Shigella* (at the taxa level ↑ *Candida* (at the genus level) ↑ *Bacteroidaceae*, *Clostridium* spp., and *Sutterella* spp ↓α diversity ↓ *Ruminococcaceae*	Strati et al., 2016; Borghi et al., 2017
Down syndrome	*Mankind*	↑ *Parasporobacterium* spp. and *Sutterella* spp. (at the family level) ↓ *Veillonellaceae* (at the family level) *Sutterella* abundance correlates with the behavioral score	Biagi et al., 2014
Parkinson’s disease	*Animal models*	Mice overexpressing the human wild-type α-synuclein exhibit constipation and impaired colonic motor function.	Wang et al., 2008
		Mice overexpressing the human α-synuclein show ↓ level of *Verrucomicrobiae.*	Gorecki et al., 2019
		Rotenone-treated mice model display an increase of the ratio of *Firmicutes*/*Bacteroidetes* phyla.	Yang et al., 2017
	*Mankind*	Abdominal pain, bloating, and constipation, irritable bowel syndrome-like bowel symptoms.	Fasano et al., 2015; Georgescu et al., 2016; Mertsalmi et al., 2017; Santos et al., 2019
		↑ *Christensenella*, *Catabacter*, *Lactobacillus*, *Oscillospira*, *Bifidobacterium*, *Christensenella minuta*, *Catabacter hongkongensis*, *Lactobacillus mucosae*, *Ruminococcus bromii*, and *Papillibacter cinnamivorans* ↑ *Escherichia coli* ↓ *Bifidobacterium* species ↓ *Dorea*, *Bacteroides*, *Prevotella*, *Faecalibacterium*, *Bacteroides massiliensis*, *Stoquefichus massiliensis*, *Bacteroides coprocola*, *Blautia glucerasea*, *Dorea longicatena*, *Bacteroides dorei*, *Bacteroides plebeus*, *Prevotella copri*, *Coprococcus eutactus*, and *Ruminococcus callidus*	Forsyth et al., 2011; Bedarf et al., 2017; Mertsalmi et al., 2017; Minato et al., 2017; Petrov et al., 2017
		↑ lytic *Lactococcus* phages ↓ *Lactococcus* bacteria	Tetz et al., 2018
Alzheimer’s disease	*Animal models*	↑*Verrucomicrobia* and *Proteobacteria* ↓ *Ruminococcus* and *Butyricicoccus*	Zhang et al., 2017
	*Mankind*	↑ *Proteobacteria* and *Bacterioidetes* (at the phyla level) ↑ Trimethylamine N-oxide (TMAO) level in the cerebrospinal fluid ↓ *Firmicutes* and *Actinobacteria* (at phyla level)	Vogt et al., 2017, 2018
Depression	*Animal models*	-The bulbectomy-induced chronic depression mouse model shows altered intestinal microbial profile. -The chronic social defeat stress (CSDS) mouse model of depression shows ↑ *Desulfovibrionaceae*, *Rikenellaceae*, *Lachnospiraceae* families, and ↓ *Allobaculum* and *Mucispirillum*	Park et al., 2013; Aoki-Yoshida et al., 2016
	*Mankind*	Irritable bowel syndrome symptoms	Sibelli et al., 2016
		Alterations in the abundance of different genera within *Bacteroidetes*, *Firmicutes*, *Proteobacteria* and *Actinobacteria* phyla. -Overall decrease in gut microbiota richness and diversity.	Kelly et al., 2016; Winter et al., 2018

### Gut Brain in Brain Development and Neurodevelopmental Disorders

The adult brain is the result of complex neurodevelopmental processes involving genetic, molecular, and environmental factors. Early during brain development, neural progenitor cells undergo cell division, and once committed to a neural fate, they migrate and differentiate into their final location in the brain making contact with their final targets. During postnatal life, experience refines the immature neuronal network during time windows of plasticity, called critical periods, when experience provides essential input for the proper refinement and final maturation of the adult brain functions. These time windows are “critical,” because if an incorrect experience occurs, it will permanently affect the structure and function of the brain (Deidda et al., 2014, 2021). So far, critical periods for the maturation of sensory systems and language productions (among others) have been described (Berardi et al., 2000; Hensch, 2004; Erzurumlu and Gaspar, 2012; Levelt and Hubener, 2012). Physiological brain development will result in normal adult brain functions; on the other hand, defects in brain development during (mal)developmental critical periods result in neurodevelopmental disorders [including autism, Rett syndrome, Down syndrome (DS), and schizophrenia] that feature main cognitive impairments (Deidda et al., 2014, 2021).

Little is still known about the role of the gut microbiome in normal brain development and to which extent it is mainly involved in neurodevelopmental disorders, but it should not come as a surprise that microbes do affect the developing brain, since the nervous system developed and had evolutionary evolved in the presence of signals from the microbes. Surely, the gut microbiota is essential for normal brain development and maturation of normal brain functions into adulthood as demonstrated in animals with absent microbiota throughout life (germ-free) that show alterations in cognition, social behavior, and stress response (Cryan and Dinan, 2012). The fact that some of these defects can be corrected by an early exposure to the microbiota (Clarke et al., 2013) suggests that there is a “critical period” for the effects on the brain to take place upon microbe exposure. Taking an example from the invertebrate animal kingdom, the development of the light-emitting organ of the Hawaiian bobtail squid critically depends on the entry of the bacterium *Vibrio fischeri* inside the organ during a critical period during development, namely, within minutes from hatching (Altura et al., 2013).

Microbiota impact on brain development starts already *in utero* and continues in the postnatal life. Prenatally (*in utero*), although the womb is considered to be sterile, the maternal microbiota can influence fetal brain development by means of metabolites reaching the fetus (Gomez de Aguero et al., 2016; Li et al., 2020). Eventually, the indirect effects of the maternal microbiota *via* its metabolites may prepare the fetus for the direct exposure to microbes at delivery. Studies performed in germ-free animals showed that maternal microbiota influences the *in utero* development of (i) the BBB as fetus gestating in pregnant germ-free mice have reduced expression of endothelial tight junction proteins and increased BBB permeability (Braniste et al., 2014); (ii) brain’s innate immune system as microglia cells are more numerous and with more branches in germ-free embryos than in controls (Thion et al., 2018); (iii) thalamo-cortical axon growth (Vuong et al., 2020); and (iv) sympathetic nerve projections to the heart (this control occurs *via* a mechanism dependent on SCFAs) (Kimura et al., 2020). Thus, maternal microbe-dependent metabolites affect offspring neural development already *in utero*.

Soon after birth, by means of the first swallow, the mammalian fetus is in contact with a world populated by microbes coming both from the mother and from the environment; next, by means of the first breast-feeding (and daily after birth) the newborn will ingest millions of bacteria contained in the breast milk (Le Doare et al., 2018). These microbes rapidly colonize the newborn in all body sites and reach the gut within a few hours where the system is already in place to convey information from gut microbiota to the brain. In fact, the connections from the vagus nerve to the duodenum and the distal small intestine already occurred *in utero*: in mice at embryonic days E14 and E16, respectively (Ratcliffe et al., 2011). In the mouse, the first microbes to colonize the guts are *Streptococcus* 1 day after birth, followed by *Lactobacillus* (required for fermenting milk lactose) by day 3 (Pantoja-Feliciano et al., 2013); this balance will shift around weaning to a more stable microbial community more dominated by *Bacteroides*. Within the 12–14 h from birth, the microbiota controls neuronal cell death (apoptosis) (Castillo-Ruiz et al., 2018), a process that massively occurs early during the first postnatal life and that sculpts the brain; the control on apoptosis was not seen prenatally, suggesting that the direct exposure to the microbes is essential. In humans, a similar sequence of gut microbe colonization occurs, but by the 1st week of life *Bifidobacterium* became more dominant (Fanaro et al., 2003).

If a normal brain development results in normal brain functions and behavior, defective brain developments have been associated with a number of neurodevelopmental disorders, including autism spectrum disorders (ASDs), Rett syndrome, Down syndrome (DS), and schizophrenia spectrum disorders (SSDs) (Di Cristo, 2007; Deidda et al., 2014, 2021). ASD is a heterogeneous neurodevelopmental disorder characterized by deficits in social communication, social interaction, and restricted/repetitive behavioral patterns. In humans, GI symptoms represent a common comorbidity in ASD (Molloy and Manning-Courtney, 2003) and the gut microbiota profile is altered in children with ASD (Cao et al., 2013) showing higher levels of *Desulfovibrio* species and *Bacteroides vulgatus*, higher levels of SCFA (Finegold et al., 2010; Wang et al., 2012), and higher levels of *Clostridium bolteae* (Pequegnat et al., 2013) compared to controls. In animal models, Buffington et al. (2016) reported that maternal high-fat diet in mice (resulting in maternal obesity during pregnancy) induced a shift in microbial composition that negatively impacts offspring social behavior (associated with increased risk of ASD). Interestingly, both social deficits and gut microbiota dysbiosis could be prevented by co-housing with offspring of mothers housed on a regular diet. Moreover, the selective re-introduction of *Lactobacillus reuteri* restored social deficits (Buffington et al., 2016), suggesting that probiotic treatment might be an effective strategy to ameliorate specific social behavioral deficits associated with neurodevelopmental disorders. Since ASD is associated with defect in cortical development (heterotopia) (Piven et al., 1990; Guerrini and Dobyns, 2014; Moffat et al., 2015), Kim et al. (2017) found out that maternal gut bacteria promote neurodevelopmental abnormalities *via* activation of the immune response in the offspring (Kim et al., 2017), possibly suggesting that some gut microbiota profiles might promote neurodevelopmental disorders.

Rett syndrome is an X chromosome-linked dominant neurodevelopmental disorder (the most prevalent in females) sharing features with ASD. Ninety-five percent of Rett syndrome cases are associated with pathogenic variants in the MECP2 (Methyl CpG Binding Protein 2) gene, encoding a chromatin-associated protein (Amir et al., 1999; Boggio et al., 2010). Main neurological symptoms include impaired language production and stereotypic hand movements. Most girls with Rett syndrome also experience epileptic seizures (Jian et al., 2006). Gastrointestinal and nutritional problems are also a common condition throughout life including pain, constipation, and chewing difficulties (Motil et al., 2012). The studies investigating gut microbiota dysfunctions in Rett syndrome are very limited with only few so far, but still clearly indicating gut microbiota dysfunctions. In the first study (Strati et al., 2016), the authors showed dysbiosis in terms of relative abundances of both the bacterial and fungal component of the gut microbiota. In particular, gut microbiota in the Rett syndrome cohort was dominated by microbial taxa belonging *to Bifidobacterium*, *Clostridia* (including *Anaerostipes, Clostridium XIVa*, and *Clostridium XIVb*) as well as *Erysipelotrichaceae*, *Actinomyces*, *Lactobacillus*, *Enterococcus*, *Eggerthella*, *Escherichi*a/*Shigella*, and the fungal genus *Candida*. Importantly, and contrary to what was previously expected, the alterations of the gut microbiota did not depend on the constipation status. In the second study (Borghi et al., 2017), the authors found (i) a lower α diversity; (ii) an enrichment in *Bacteroidaceae*, *Clostridium* spp., and *Sutterella* spp.; and (iii) a slight depletion in *Ruminococcaceae* in girls and women with Rett syndrome.

Down syndrome (DS) is the most frequent cause of intellectual disability; it is caused by the trisomy of human chromosome 21 and it presents multiple GABA dysfunctions in animal models and humans (Bittles et al., 2007; Dierssen, 2012; Deidda et al., 2015; Contestabile et al., 2017). One study had investigated the DS gut microbial community in adult (Biagi et al., 2014). Although DS gut microbiota did not differ from healthy control in the relative abundance of the dominant microbial families (i.e., *Ruminococcaceae*, *Lachnospiraceae*, *Clostridiales*, *Bifidobacteriaceae*, and *Bacteroidaceae*), *Parasporobacterium* spp. and *Sutterella* spp. were increased, whereas *Veillonellaceae* was reduced. Interestingly, *Sutterella* abundance was positively correlated with the aberrant behavior score (Biagi et al., 2014).

Schizophrenia spectrum disorder (SSD) is a heterogeneous neurodevelopmental disorder characterized by positive symptoms (namely, symptoms that involve any change in normal behavior; they arise with the pathology, for example, delusions and hallucinations), negative symptoms (involving an impairment in normal behavior; for example lack of interest in social interactions, affective flattening, and avolition), and cognitive symptoms. Psychotic symptoms usually manifest clinically during the adolescent period. The gut microbiota was proposed to have an impact on social cognition affecting SSD (Dinan et al., 2014) and several clinical trials investigated microbiota profile differences between people with SSD and controls. Schwarz et al. (2018) investigated the gut microbiota in people with first episode psychosis (FEP) and found an increase in *Lactobacillus*, *Saccharophagus*, *Ochrobactrum*, *Tropheryma*, *Halothiobacillus*, *Deferribacter*, and *Halorubrum* (at the genus level), and a decrease in *Anabaena*, *Nitrosospira*, and *Gallionella* in comparison to healthy controls. The number of *Lactobacillus* bacteria correlated positively with severity of psychotic symptoms (Schwarz et al., 2018). Castro-Nallar et al. (2015) analyzed the oropharyngeal microbiome finding an increase in the numbers of *Lactobacillus*, *Bifidobacterium*, and *Ascomycota* in people with SSD (Castro-Nallar et al., 2015). When analyzing the gut fungal composition (the mycobiome), Severance et al. (2016) found an increase in *Candida albicans* specifically in male with SSD, not in females (Severance et al., 2016) calling for possible probiotic treatment interventions.

Altogether, the findings reviewed so far clearly show how the gut microbiota plays a role in normal brain development (already *in utero*), and how neurodevelopmental disorders are characterized by major gut microbiota dysbiosis. So far, despite the fact that these findings suggest that the link between neurodevelopmental disorders and gut microbiota is correlative rather than causal, probiotic strategies aimed to reinstate physiological microflora balance might be a promising new path to be undertaken for alleviating both GI and eventually core symptoms in people with neurodevelopmental disorders.

### Gut Brain in Parkinson’s Disease

Parkinson’s disease is the second most common neurodegenerative disorder of aging and it affects more than 6 million people in the world (Disease et al., 2016). It results primarily from the death of dopaminergic neurons in the substantia nigra pars compacta, a basal ganglia structure located in the midbrain and involved in the control of movements (Moore et al., 2005). The neuropathological hallmark in Parkinson’s disease is the presence of Lewy bodies, namely, cytoplasmic inclusions of α-synuclein aggregates in the neurons of the substantia nigra pars compacta (Okazaki et al., 1961; Polymeropoulos et al., 1997) whose soluble oligomeric conformations is thought to mediate the toxic effects in the pathology (Miller et al., 2021).

People with Parkinson’s disease display a major impairment in their motor behavior showing characteristic symptoms including tremor at rest, rigidity, and slowness in starting the movement (bradykinesia) (Antony et al., 2013). Moreover, non-motor symptoms become common in the advanced stages of the disease, and they include cognitive impairment (dementia), psychosis, apathy, restlessness, impulse control disorders, sleep disorders, and associated comorbidities (depression and anxiety) (Stacy, 2011; Antony et al., 2013; Pfeiffer, 2016). Moreover, olfactory dysfunctions (Haehner et al., 2009; Fullard et al., 2017), pain, and sensory disturbances (Chen Y. et al., 2015; Tseng and Lin, 2017) are common. These non-motor symptoms have, in particular, been considered valuable for the early diagnosis of Parkinson’s disease during the so-called “premotor phase” of the disease because they can occur years before the onset of the motor symptoms (Chen H. et al., 2015; Rizzo et al., 2016).

Among the pre-motor events, Braak et al. (2003) previously postulated that lesions in the ENS that occurred at a very early stage of the disease could represent a route of entry for a putative exogenous “unknown pathogen” that passes across the gastric mucosa and then is retrogradely transported up the vagus nerve to the medulla to initiate the pathological processes leading to Parkinson’s (Braak et al., 2003). Following this interesting hypothesis, hard and solid data are still missing and evidence in the literature show contradicting results. For example, one study in humans showed no statistically significant association between *T. gondii* infection and idiopathic Parkinson’s disease (Celik et al., 2010), while another one in mice showed a significant increase in dopamine metabolism in neural cells (Prandovszky et al., 2011). A very recent study challenged Braak’s hypothesis revealing α-synuclein aggregates in the vagus nerve and in the stomach of people with Parkinson’s disease, but not in normal elderly subjects, thereby supporting initiation of α-synuclein pathology in the brain and is thereby against the “Body-First” hypothesis (Beach et al., 2021).

Independently whether the latter hypothesis would be confirmed or not, nowadays, it is widely accepted that among the non-motor symptoms, the GI ones are common among people with Parkinson’s disease (Fasano et al., 2015; Santos et al., 2019) who report abdominal pain, bloating, and constipation (Georgescu et al., 2016) even before being diagnosed with the disease (Abbott et al., 2001). Moreover, IBS-like bowel symptoms have been reported and, importantly, connected with a disruption of the gut microbiota linked to a lower abundance of *Prevotella* species in patient fecal samples (Mertsalmi et al., 2017). Lower *Bifidobacterium* species counts were also found (Minato et al., 2017).

Plenty of evidence in the literature indicates gut microbiome dysfunctions in people with Parkinson’s disease (Hamano et al., 1993; Clairembault et al., 2015; Hasegawa et al., 2015; Keshavarzian et al., 2015; Klingelhoefer and Reichmann, 2015; Mulak and Bonaz, 2015; Unger et al., 2016; Bedarf et al., 2017; Hill-Burns et al., 2017; Parashar and Udayabanu, 2017; Scheperjans et al., 2018; Sun and Shen, 2018; Breen et al., 2019; Santos et al., 2019) with changes in the microbiome composition of the upper (oral) GI tract occurring at the early stage of the disease (Mihaila et al., 2019), suggesting that probiotics/psychobiotics and microbiota transplantation might be beneficial (Dinan and Cryan, 2015). Apart from GI disorders, changes in the gut bacterial abundances of microbes (such as *Prevotellaceae* and *Enterobacteriaceae*) (Scheperjans et al., 2015), activation in the brain of microglial cells by short-chain fatty acids (SCFA), and intestinal flora metabolites have been reported (Erny et al., 2015).

Aggregates of α-synuclein were described in the ENS of people with Parkinson’s disease (Barrenschee et al., 2017), and recent evidence obtained in animal models (rat and *Caenorhabditis elegans*) showed that alterations in the gut microbiome may in fact induce accumulation of α-synuclein aggregates in the ENS (Chen et al., 2016). In a very interesting study, Sampson et al. (2016) demonstrated that the gut microbiota might play a sufficient role for the onset of the disease. The authors demonstrated in mice overexpressing α-synuclein that (i) the presence of gut microbiota is necessary to promote Parkinson-like pathological alterations, and that (ii) transplantation of fecal samples derived from people with Parkinson’s disease into normal mice resulted in impaired motor function, suggesting that microbes in the gut may play a pivotal role in the onset of Parkinson’s disease (Sampson et al., 2016). Moreover, mice overexpressing the human wild-type α-synuclein exhibit constipation and impaired colonic motor function (Wang et al., 2008).

The microbiota pattern in people with Parkinson’s disease can trigger local inflammation followed by aggregation of α-synuclein and generation of Lewy bodies exhibiting thereby a pro-inflammatory profile; the changes in the microbiota content includes 9 genera and 15 species of microorganisms: (i) a reduced content of *Dorea*, *Bacteroides*, *Prevotella*, *Faecalibacterium*, *Bacteroides massiliensis*, *Stoquefichus massiliensis*, *Bacteroides coprocola*, *Blautia glucerasea*, *Dorea longicatena*, *Bacteroides dorei*, *Bacteroides plebeus*, *Prevotella copri*, *Coprococcus eutactus*, and *Ruminococcus callidus*, and (ii) an increased content of *Christensenella*, *Catabacter*, *Lactobacillus*, *Oscillospira*, *Bifidobacterium*, *Christensenella minuta*, *Catabacter hongkongensis*, *Lactobacillus mucosae*, *Ruminococcus bromii*, and *Papillibacter cinnamivorans* (Bedarf et al., 2017; Petrov et al., 2017). The resulting increased intestinal permeability (gut leakiness) to proinflammatory bacterial products in the intestine correlated with the increased intestinal mucosa staining for *Escherichia coli* bacteria, nitrotyrosine, and α-synuclein, markers in early phases of Parkinson’s disease (Forsyth et al., 2011), giving strength to the view that the intestine might be an early site of Parkinson’s disease in response to an environmental toxin or pathogen. Also, bacterial amyloids may favor pro-inflammatory environment in the intestine (Miraglia and Colla, 2019). In rats, lipopolysaccharide (LPS) intranigral injection induces inflammatory reaction that damages the nigrostriatal dopaminergic system, indicating that this event may be indeed implicated in the neurodegeneration processes (Castano et al., 1998). Gorecki et al. (2019) further explored the interaction between the gut microbiota and α-synuclein aggregation investigating the effects of LPS in mice overexpressing the human α-synuclein. By using 16S ribosomal RNA sequencing, the authors first found out that the relative abundance of mucin-degrading *Verrucomicrobiae* and LPS-producing Gammaproteobacteria were greater in fecal samples from people with Parkinson’s disease, while in mice overexpressing the human α-synuclein *Verrucomicrobiae* were reduced. Then, they investigated the effect of LPS on intestinal barrier function *in vitro* and found out that LPS exposure reduced and altered the distribution of the cell membrane tight junctions. Moreover, *in vivo* LPS administration resulted in the emergence of early motor manifestations in mice overexpressing the human α-synuclein, supporting the concept that proinflammatory gut microbiome environment might be a trigger for Parkinson pathogenesis (Gorecki et al., 2019). Despite being extensively described in Parkinson’s disease, the neuropathological role of inflammation is common among other neurodegenerative diseases too (Cappellano et al., 2013).

In studies performed in rodents, gut microbiota transplants from a mouse model of Parkinson’s disease induced motor impairment and striatal neurotransmitter decrease into normal mice and fecal microbiota transplantation (FMT) reduced the activation of microglia and astrocytes in the substantia nigra, reduced gut microbial dysbiosis, decreased fecal SCFAs, alleviated physical impairment, and increased striatal dopamine and serotonin content in a mouse model of Parkinson’s disease (Sun et al., 2018).

As far as phage-related dysbiosis in Parkinson’s disease is concerned, evidence in the literature suggests that phages might be implicated in the misfolding of the α-synuclein (Tetz and Tetz, 2018) and that the lytic bacteriophages in the gut might contribute to the onset of the pathology. In fact, the lytic *Lactococcus* phages are more abundant in people with Parkinson’s disease than in healthy individuals, and it is associated with a strong reduction in *Lactococcus* bacteria (Tetz et al., 2018); since *Lactococcus* bacteria play an important role in producing the neurotransmitter dopamine (Strandwitz et al., 2019), and do regulate the permeability of the gut (Darby et al., 2019) [dopamine and gut permeability are two factors linked to the early signs of Parkinson’s Disease in the gut (Houser and Tansey, 2017)] The depletion of *Lactococcus* bacteria caused by the high numbers of strictly lytic phages in people with Parkinson’s disease might play a role in triggering α-synuclein misfolding (Braak et al., 2003; Asano et al., 2012; Scheperjans, 2016).

Mitochondrial dysfunctions (including impairment in the mitochondrial electron transport chain, alterations in the mitochondrial morphology, and mutations in mitochondrial DNA) also play a role in the pathophysiology of Parkinson’s disease occurring early in the pathogenesis (both in the sporadic and monogenic forms of the disease) and leading to impairments in energy production, generation of reactive oxygen species, and induction of stress-induced apoptosis (Subramaniam and Chesselet, 2013). Consequently, mitochondria represent a valuable target for neuroprotective interventions.

Despite the fact that gut microbiota dysfunctions in Parkinson’s disease are well documented, it is unclear whether the changes in the intestinal microflora are either a cause or an effect of the disease. The study led by Yang et al. (2017) showed that it might be a cause of the pathology. In fact, in a rotenone-treated mouse model, alteration of fecal microbiota compositions (an overall decrease in bacteria diversity and changes of microbiota composition with an increase in the ratio of *Firmicutes*/*Bacteroidetes* phyla) precedes the onset of α-synuclein pathology (Yang et al., 2017). In humans, the intestinal flora changes as the pathology progresses, and these changes do correlate with the clinical symptoms of the disease (Li et al., 2017).

Taken together, gut microbiota shows profound dysbiosis in Parkinson’s disease, as shown in humans and in animal models, and by correcting this dysbiosis would open new therapeutic avenues for the disease.

### Gut Brain in Alzheimer’s Disease

Alzheimer’s disease is the most common form of dementia in the elderly, with main medical and social problems in economically developed countries connected to a significant increase in human life span. It is characterized by an irreversible neurodegeneration impacting learning and memory functions, ultimately leading to disability (Wimo et al., 2017). The prevalence rate of the disease as well as the degree of cognitive impairment are influenced by factors like older age, low education level, and poor health conditions (de Souza-Talarico et al., 2016). During the progression of the disease, neuronal death leads to progressive impairments in synaptic function. Neurodegeneration also strikes catecholaminergic neurons located in the locus coeruleus (LC) that provides dense innervations to the thalamus and amygdala, and sparse innervations of the neocortex, hippocampus, cerebellum, and spinal cord. Similarly to Parkinson’s disease where α-synuclein forms aggregates, Alzheimer’s disease neuropathology is characterized by deposits of extracellular amyloid-β (Aβ) peptide and intraneuronal neurofibrillary changes of hyper-phosphorylated protein tau (Braak and Braak, 1991; Tiraboschi et al., 2004). Aβ peptides are produced by proteolytic cleavage of the transmembrane amyloid precursor protein (APP), whereas tau is a brain-specific and axon-enriched protein normally associated with cytoskeleton microtubules. Upon phosphorylation, tau loses its affinity with the microtubules and destabilizes the neuronal cytoskeleton leading to synaptic neuronal deficiency, disruption of Ca^2+^ homeostasis and ultimately neuronal death *via* apoptosis (Yankner et al., 1990; Hardy and Higgins, 1992). Despite the extensive preclinical/clinical research and pharmacological development, the precise mechanisms underlying Alzheimer’s disease are unclear, and the current therapeutic approaches targeting Aβ deposits are able to only provide modest symptom relief (Bachurin et al., 2018). Apart from genetic factors, oxidative stress represents one of the mechanisms where the pathological intra-/extracellular deposits mediate the neurodegenerative process, including an inadequate antioxidant system, high oxygen consumption, excitotoxic amino acids, and high iron content, altogether promoting the production of reactive oxygen (ROS) and nitrogen species (RNS) (Kim et al., 2015).

Similar to Braak’s hypothesis for Parkinson’s disease where an external pathogen would cause neuroinflammation [described earlier in the text (Braak et al., 2003)], pathogen-induced inflammation might also play a role in Alzheimer’s disease (Wyss-Coray and Rogers, 2012; Cappellano et al., 2013); for example *T. gondii*, which is an obligate intracellular parasitic protozoan (phylum Apicomplexa) that uses a variety of mammals as intermediate hosts (including humans), with the cat as the final one, causes the infectious disease toxoplasmosis (da Silva and Langoni, 2009). It has been described that it can induce immune response in the host and inflammation in the CNS, alteration in the levels of neurotransmitters, and direct infections of neuronal and astrocytic cells (in rat primary cell culture) (Luder et al., 1999). For these reasons, *T. gondii* can be considered a possible etiologic risk factor for Alzheimer’s disease (Nayeri et al., 2021).

Apart from *T. gondii*, a number of other infectious agents may be involved in Alzheimer’s disease including viral [i.e., Herpes simplex virus-1 (HSV-1) (Itzhaki et al., 1997); Herpes simplex virus type 2 (HHV-2) (Kristen et al., 2015); cytomegalovirus (CMV) (Renvoize et al., 1979; Renvoize and Hambling, 1984); hepatitis C virus (HCV) (Chiu et al., 2014)], fungal [i.e., *Candida famata*, *C. albicans*, *Candida glabrata*, and *Syncephalastrum racemosum* (Pisa et al., 2015a,b)], and bacterial [i.e*., Borrelia burgdorferi* (MacDonald and Miranda, 1987); *Chlamydia pneumonia* (Paradowski et al., 2007; Lim et al., 2014); *Treponema pallidum* (Riviere et al., 2002); and *Helicobacter pylori* (Kountouras et al., 2009)].

It is surprising to consider that the intracellular Aβ production and deposition in Alzheimer’s disease could be a consequence (rather than the cause) set in motion to counteract and protect from the pathogen invasion in the CNS. Indeed, several types of spirochetes have been associated to dementia, cortical atrophy, and biological hallmarks of the disease; neurons infected by HSV-1 showed Aβ and hyperphosphorylated tau accumulation, and Aβ displayed anti-microbial properties capable of inducing pore formation, thus justifying their infection-mediated accumulation (Ashraf et al., 2019).

As far as microbial communities residing in the gut are concerned, recent studies have implicated the gut microbiome as another etiological factor involved in the pathogenesis of Alzheimer’s disease both in humans and in animal models (Harach et al., 2017; Vogt et al., 2017; Zhang et al., 2017; Spielman et al., 2018; Ashraf et al., 2019; Kowalski and Mulak, 2019; Li et al., 2019). In humans, the composition of the intestinal microbiome differs between people with Alzheimer’s disease and healthy controls, including a decreased numbers of *Firmicutes* and *Actinobacteria* and an increase in the number of bacteria belonging to the *Proteobacteria* and *Bacterioidetes* phyla (Vogt et al., 2017). Interestingly, Gammaproteobacteria, Enterobacteriales, and Enterobacteriaceae of phylum Proteobacteria that are known to induce inflammation (“pro-inflammatory bacteria”) increased steadily from healthy controls to mild cognitive impairment and dementia stage (Vogt et al., 2017). Moreover, microbiota-derived metabolic molecule trimethylamine N-oxide (TMAO, a small molecule produced by the metabolism of dietary choline) were detected in the cerebrospinal fluid of people with Alzheimer’s disease and associated with biomarkers of Alzheimer’s pathology (i.e., phosphorylated tau and phosphorylated tau/Aβ42) (Vogt et al., 2018). Interestingly, the microbiota alterations might arise earlier than Alzheimer’s clinical symptoms as suggested by a study that shows similar alterations in gut microbiota composition between people with mild cognitive impairment and Alzheimer’s disease (Li et al., 2019); thus, it can be considered as an early presymptomatic event as already suggested for Parkinson’s disease. In animal models, (i) in the Aβ precursor protein (APP) transgenic mouse model, APP-mutant germ-free mice displayed decreased cerebral Aβ amyloid pathology in comparison to APP mice in control conditions, and anti-Aβ effects could be blocked by reconstruction of these germ-free APP mice with microbiota derived from conventional mice (Harach et al., 2017); (ii) long-term broad-spectrum antibiotic treatment reduces Aβ deposition and improves the neuropathological phenotype in the APPSWE/PS1DeltaE9 mouse model (Minter et al., 2016); (iii) fecal samples derived from another model of the disease showed dramatic elevations in *Verrucomicrobia* and *Proteobacteria*, as well as significant reductions of *Ruminococcus* and *Butyricicoccus*, suggesting a pathological relevant microbiota composition and diversity (Zhang et al., 2017). Activated microglia also contribute to the pathology of AD by inhibiting Aβ clearance and increasing Aβ deposition (Cai et al., 2014).

Taken together, the evidence reviewed here clearly indicates that specific species of gut microbiota activate Aβ signaling pathways and contribute to the pathogenesis of Alzheimer’s disease. Consequently, modification of the gut microbiota composition by probiotic supplementation may create new preventive and therapeutic options for interventions in people with Alzheimer’s disease.

### Gut Brain in Depression

Depression is a common and heterogeneous disorder responsible for significant disability worldwide, and it is characterized by a state of low mood often accompanied with loss of interest in activities that the individual normally perceives as pleasurable. Its severe form, major depression, is classified as a mood disorder. Depression is a leading cause of disability worldwide, it has greater negative effects on people health (Moussavi et al., 2007), and it carries a similar risk for mortality akin to smoking, blood pressure, and alcohol intake (Mykletun et al., 2009). It is an associated comorbidity in epilepsy (Stafford-Clark, 1954; Harden, 2002) and in major neurodegenerative disorders, and its etiology includes metabolic (Jokela et al., 2014), neuroendocrine (Stetler and Miller, 2011), and neuroimmune factors (Dowlati et al., 2010).

As for the other brain diseases discussed so far (Parkinson’s and Alzheimer’s diseases) although depression is primarily a disease of the brain, the brain does not exist in isolation, but it is embedded within the overall physiology of the body, including the gut (Valles-Colomer et al., 2019). If the gut microbiota of adult healthy individuals is primarily dominated (90%) by *Bacteroidetes* and *Firmicutes* phyla, the gut microbiota of people with major depressive disorder displays a significant alteration in the abundance of different genera within *Bacteroidetes*, *Firmicutes*, *Proteobacteria*, and *Actinobacteria* phyla (Winter et al., 2018). In order to explore the relation between depression and microbiota, Kelly et al. (2016) investigated in humans and animal models whether changes in the gut microbiota composition and function might mediate the dysregulation of the neuroimmune and neuroendocrine pathways, both involved in depression. The authors assessed the cytokines, salivary cortisol and plasma LPS binding protein, and collected fecal samples from both groups to determine the microbiota profiles; they found that clinical depression was associated with decreased gut microbiota richness and diversity. Next, they obtained fecal microbiota samples derived from either people with major depression or controls, and performed FMT by means of oral gavage in a microbiota-deficient rat model. Strikingly, FMT from people with depression induced behavioral and physiological features characteristic of depression, including anhedonia and anxiety-like behaviors (Kelly et al., 2016).

Depression often coexists with IBS (Sibelli et al., 2016), which is characterized by alterations in gut function, and data obtained from animal studies show that the gut microbiota may affect the neurobiological features of depression. Park et al. (2013) exploited the bilateral olfactory bulbectomy in mice, known to induce depression-like behavior, to investigate whether it would result in changes in the microbiota composition: the bulbectomy-induced chronic depression resulted in an altered intestinal microbial profile together with an increase in colon motility, c-Fos activity, and serotonin levels (Park et al., 2013). Since depression is a common symptom in people with obesity (Bruce-Keller et al., 2009), Bruce-Keller et al. (2015) investigated whether the transplanted microbiome from obese mice (that show depression-like behavior) into non-obese control mice would result in depressive-like behavior. Before the transplantation, control mice were fully depleted of their own microbiome. Obese-derived microbiome re-colonization in control non-obese mice resulted in a disruption in exploratory, cognitive, and stereotypical behavior in the absence of significant differences in body weight (Bruce-Keller et al., 2015).

Depressive-like behaviors can also be induced in mice by means of stress models, for example, that of the chronic social defeat stress (CSDS) paradigm where mice are repeatedly subjected to social defeat by a larger and more aggressive mouse. Stressed mice developed depressive-like behaviors and showed changes in microbial diversity with an increase in *Desulfovibrionaceae*, *Rikenellaceae*, and *Lachnospiraceae* families and a decrease in *Allobaculum* and *Mucispirillum* (Aoki-Yoshida et al., 2016).

This evidence suggests that the gut microbiota may play a causal role in the development of depression and may be considered a valuable target in the treatment/prevention of this disorder.

## Microbiota-Targeted Strategies: From Phage Therapy to Pre/Probiotic Approaches for Brain Pathologies

The concept that gut microbiota affects not only the gut, but also the brain, and that brain–gut communication dysfunctions might play a role in the pathogenesis of neurological psychiatric disorders brought general interest in the search for new therapeutic solutions to tackle these pathologies. In particular, the use of pre- or probiotics (live microbes) aimed to correct the dysbiosis and/or microbiota defects and ameliorate the pathophysiological condition came into the spotlight recently.

When thinking about strategies to control/restrict uncontrolled/pathogenic gut microbial populations, the *antimicrobial therapy* (aimed to target directly the microbes) surely comes to mind. The antimicrobial therapy indeed firstly came into the spotlight, but it lost scientific and therapeutic interest over recent decades because of both the lack of development of new anti-bacterial agents and the widespread antimicrobial resistance, the latter alone being considered to be a great challenge to the health of the entire population because it results in 700,000 deaths every year, and, if not tackled properly with innovative strategies, “would lead to 10 million deaths by 2050, exceeding the toll of cancer” (Gorski et al., 2016). Consequently (and necessarily), the antimicrobial strategy was overcome by an alternative *phage therapy* that exploits the use of bacteria-specific viruses (phages) (Hyman and Abedon, 2010) to combat populations of uncontrolled or pathogenic bacteria (Chan et al., 2013; Gorski et al., 2016), for example, against multi-drug resistance *Staphylococcus aureus* (Gupta and Prasad, 2011). Generally speaking, phage therapy has multiple strengths in comparison to chemical antibiotics (used in the antimicrobial therapy), and it has limitations as well. Among the advantages, phages are effective bactericidal agents thereby limiting the viability of infected bacteria, contrarily to certain antibiotics that, being bacteriostatic rather than bactericidal, might still allow the evolution toward antibiotic resistance (Stratton, 2003). Also, the narrow host range (Hyman and Abedon, 2010) limits *per se* the number of bacterial types that could achieve phage resistance. Moreover, phages are easily discovered (often from sewage or waste materials); they are basically non-toxic, being composed of nucleic acids and proteins (Abedon and Thomas-Abedon, 2010), and they specifically increase in number where the bacterial host is located (“auto dosing”) (Carlton, 1999). Again, thanks to their specific host selectivity (Hyman and Abedon, 2010), phages exert a minimal disruption of normal flora (Skurnik et al., 2007). Finally, since phages amplify themselves, they can be applied with a single dose, already achieving active therapy (Abedon and Thomas-Abedon, 2010). The single dose (or less frequent doses) consequently increases the safety and reduces the environmental impact of phage therapy (Abedon and Thomas-Abedon, 2010). Phage therapy comes with disadvantages and limitations. Indeed, not all phages meet the criteria for being good therapeutics (Abedon and Thomas-Abedon, 2010), thereby limiting the bacterial strains that can be targeted. Moreover, they can interact with the immune system and release bacterial components (including toxins) while killing bacteria, resulting in harmful immune responses. In addition, the medical establishment is mostly unfamiliar with phages and few phage product passed regulatory standards (Kutter et al., 2010). Finally, the general perception by the general public of phages as “viruses” might be misinterpreted and limits its exploitation in therapy being connected to viral pathogens that lead to human diseases. Although the advantages of phage therapy looked promising, so far it has been exploited more for the phages’ ability to directly reverse the aggregation of misfolded proteins in the brain [for example, in Alzheimer’s of Parkinson’s diseases (Messing, 2016)] rather than to target and correct gut dysbiosis related to brain pathologies. For example, the phage therapy might contribute to future therapeutic intervention in Parkinson’s disease, which should need to be confirmed in further studies through targeted approaches to manipulate the phagobiota. This will be based on significant alterations in the representation of certain bacteriophages in the phagobiota of people with Parkinson’s disease (Tetz et al., 2018). Nevertheless, so far, the phage therapy had not been directly exploited to modulate gut microbiota in order to ameliorate neurological conditions.

Beyond the antimicrobial and phage therapies, the FMT, which involves the transplantation of functional microbiota from healthy individuals into the GI tract of people suffering from a certain disease in order to “reconstruct” the healthy microbial composition and improve the clinical symptoms, surely represents a new therapeutic approach from the gut to the brain, and it has been investigated in a number of clinical trials (both concluded and ongoing) in multiple conditions, such as hepatic encephalopathy, ASD, Parkinson’s disease, multiple sclerosis, Alzheimer’s disease, and epilepsy [see the reviews (Evrensel and Ceylan, 2016; Xu et al., 2021)].

Studies performed in animal models showed that FMT is effective is transferring/restoring disease-associated features: (i) FMT using fecal transplants derived from normal mice into a mouse model of Parkinson’s disease reduced Parkinson-related signatures, namely, microglia and astrocyte activation in the substantia nigra, gut microbial dysbiosis, and decreased fecal SCFAs, and it alleviated physical impairment and increased striatal dopamine and serotonin content (Sun et al., 2018); (ii) FMT using fecal transplants obtained from people with major depression into normal mice induced behavioral and physiological features characteristic of depression, including anhedonia and anxiety-like behaviors (Kelly et al., 2016); (iii) FMT from normal mice to the Fmr1 KO mice (an animal model of Fragile X syndrome) ameliorated the autistic-like behaviors, especially memory deficits and social withdrawal (Goo et al., 2020).

In humans, three rounds of FMT were effective in improving (i) constipation and motor symptoms in a person with Parkinson’s disease (the improvement of motor symptoms lasted only for 2 months) (Huang et al., 2019), and (ii) neurological and GI symptoms in a 17-year-old patient with epilepsy complicated with Crohn’s disease (He et al., 2017). In children with ASD, FMT showed to be effective not only in reducing GI disorders (Zebrowska et al., 2021), but also in alleviating neurological symptoms. In fact, FMT performed in children with ASD improved the autistic behavioral symptoms together with an improvement of bacterial diversity toward a physiological state (Kang et al., 2019). The positive effects persisted for 8 weeks after the end of FMT. Another independent clinical trial showed the positive effect of FMT in ameliorating autistic-like symptoms (Zhao et al., 2019).

In particular, so far, FMT proved to be effective in ameliorating neurological symptoms in patients that underwent fecal transplantation firstly because of infections caused by *C. difficile* (Carlucci et al., 2016; Evrensel and Ceylan, 2016; Zhang et al., 2018; Vendrik et al., 2020; Xu et al., 2021). In a very recent case report, Park et al. (2021) explored the FMT strategy in a patient with Alzheimer’s disease. The patient was diagnosed with severe *C. difficile* infection and, given the failure of the antibiotics treatment, she underwent two rounds of FMT; stools were derived from a healthy donor. Following the transplantation and the eradication of the pathogen, the patient showed improvements in GI symptoms and, importantly, a slight improvement in the cognitive functions in terms of short-term memory, semantic skills, attention, and non-verbal learning, together with a marked improvement in mood and expressive affection (Park et al., 2021). A second study showed remission of *C. difficile* infection and improvement in cognitive performance in a patient with Alzheimer’s disease who underwent FMT derived from stools from his wife (Hazan, 2020). The latter two studies in humans confirmed the results obtained in a previous study obtained in a mouse model of Alzheimer’s disease that showed improved cognitive function after FMT derived from normal control (Sun et al., 2019). Altogether, these studies in humans and in an animal model gave insights into how FMT could represent a valuable tool to ameliorate both GI and cognitive symptoms in Alzheimer’s disease. Two studies examined the effect of FMT in a total of four patients with multiple sclerosis and diagnosed with *C. difficile* infection (Borody et al., 2011; Makkawi et al., 2018). FMT resolved *C. difficile* infection and constipation together with a progressive improvement in neurological symptoms. Some cases reported slow resolution of leg paresthesia and regaining the ability to walk long distances unassisted.

Despite the fact that FMT showed remarkable therapeutic features, it transfers hundreds of strains and it could thereby potentially be problematic because donors could transfer opportunistic pathogens or infections to recipients. Different from FMT, the use of probiotics, which only supplements some bacterial strains, has been shown to affect the functionality of the CNS by means of beneficial interactions with the commensal microbial gut community and by modulation of gut-derived inflammation (Wang et al., 2016). For example, since pro-inflammatory events triggered by LPS might be implicated in neurodegenerative processes [like that one of the nigrostriatal dopaminergic system in rodents (Castano et al., 1998)], studies challenged whether the administration of probiotics can attenuate LPS effects. In a first study, Rodes et al. (2013) investigated the effects of probiotics *Lactobacillus* and *Bifidobacterium* on gut-derived LPS and inflammatory cytokine concentrations using human colonic microbiota model. After 14 days of administration with either *B. bifidum*, *L. rhamnosus*, *B. longum*, and *B.* longum subsp. *Infantis*, LPS concentrations were reduced. *B. longum* subsp. *infantis* showed higher ability to reduce TNF-α concentrations and to increase IL-4 concentrations (Rodes et al., 2013). In a successive study, Musa et al. (2017) investigated in mice whether lactic acid bacteria (either *Lactobacillus fermentum* LAB9 or *L. casei* LABPC) fermented cow’s milk exhibited neuroprotective properties against the LPS-induced inflammatory effects. The authors found out that oral administration of fermented cow’s milk containing lactic acid bacteria attenuated memory deficits (assessed *via* the Morris Water Maze) in mice treated with LPS. Moreover, the treatment increased antioxidants and reduced pro-inflammatory cytokines (Musa et al., 2017). These studies showed potential applications of probiotics in the field of biotherapy. Here, it follows an extensive overview of the microbiota-targeted strategies in brain disorders using pre- and probiotics. Table 2 summarizes pre/probiotic effects in animal models and in clinical trials in people with neurological disorders.

**TABLE 2:** Pre/probiotic effects in animal models and in clinical trials in people with neurological disorders.

Brain disorder/disease		Treatment	Results	Selected references
Autism spectrum disorders	*Clinical trials*	Oral administration of the probiotic *Lactobacillus acidophilus* twice a day for 2 months.	Decrease of the aberrant urine levels of the metabolite D-arabinitol (derived from the *Candida* pathogenic-species).	Kaluzna-Czaplinska and Blaszczyk, 2012
		Supplementation of probiotics *Lactobacillus acidophilus*, *Lactobacillus rhamnosus* and *Bifidobacteria longum* for 3 months.	- Increase in the counts of *Bifidobacteria* and *Lactobacilli* levels in the stools. - Improvement in the severity of autism, gastrointestinal symptoms (compared to the pre-treatment period).	Shaaban et al., 2018
		Supplementation of the “De Simone Formulation” for 6 months. “De Simone” formulation contains eight probiotic strains: *Streptococcus thermophilus*, *Bifidobacterium breve*, *Bifidobacterium longum*, *Bifidobacterium infantis*, *Lactobacillus acidophilus*, *Lactobacillus plantarum*, *Lactobacillus para-casei*, and *Lactobacillus delbrueckii* subsp. *Bulgaricus.*	- Decrease in the severity of autistic score in the group of children without gastrointestinal symptoms. - Improvement of the gastrointestinal symptoms in the children with the latter symptoms.	Santocchi et al., 2020
Schizophrenia spectrum disorders	*Clinical trials*	Supplementation of the probiotics *Lactobacillus rhamnosus* strain GG and *Bifidobacterium animalis* subsp. *lactis* Bb12 for 14 weeks.	- Reduced *Candida albicans* antibodies in male patients. - Trend to improve the positive psychiatric symptoms.	Severance et al., 2017
Parkinson’s disease	*Animal models*	6-OHDA rat model of Parkinson’s disease treated for 6 weeks with *Lacticaseibacillus rhamnosus* HA-114.	Improved hippocampal-dependent cognition deficits.	Xie and Prasad, 2020
	*Clinical trials*	Supplementation of fermented milk containing *Lactobacillus casei Shirota* up to 3 weeks	Improved stool consistency and defecation	Cassani et al., 2011
		Probiotics in tablets *Lactobacillus acidophilus* and *Bifidobacterium infantis* twice per day for 3 months.	Improved abdominal pain and bloating.	Georgescu et al., 2016
		Probiotics containing four strains of *Lactobacillus acidophilus*, *Lactobacillus reuteri*, *Bifidobacterium bifidum*, and *Lactobacillus fermentum* for 12 weeks.	Improvement of the movement parameters assessed by means of the Movement Disorders Society-Unified Parkinson’s Disease Rating Scale (MDS-UPDRS).	Tamtaji et al., 2019
		Probiotic supplementation of *Lactobacillus reuteri* for 14 days to *H. pylori*-positive patients: This study was not focused on people with Parkinson’s disease)	- Eradication of *Helicobacter pylori* infection (known to decreased L-dopa absorption and increased motor function fluctuations)	Ojetti et al., 2012
		Peripheral blood mononuclear cells isolated from people with Parkinson’s disease treated *in vitro* with probiotic bacterial strains belonging to the *Lactobacillus* and *Bifidobacterium* genus.	- Reduced inflammatory cytokines and the production of reactive oxygen species. - Antagonized the growth of the pathogenic bacteria *Escherichia coli* and *Klebsiella pneumoniae.*	Magistrelli et al., 2019
Alzheimer’s disease	*Animal models*	Mice treated by intraventricular injection of Aβ given oral subchronic administration (11 days) of *Bifidobacterium breve* strain A1.	- Rescue of the cognitive impairment assessed by the Y maze behavioral test. - Suppression of the Aβ-induced changes in gene expression of inflammation and immune-reactive genes.	Kobayashi et al., 2017
		Triple-transgenic mice model of Alzheimer’s disease 3xTg-AD given SLAB51 probiotic formulation (including lactic acid bacteria and bifidobacteria) for 4 months in the drinking water.	- Decreased cognitive decline. - Reduced accumulation of Aβ aggregates. - Reduced oxidative stress.	Bonfili et al., 2017, 2018
	*Clinical trials*	Mixed-species probiotics supplementation that included *Lactobacillus acidophilus*, *Lactobacillus casei*, *Bifidobacterium bifidum*, and *Lactobacillus fermentum* for 12 weeks probiotic	- Improved the mini-mental state examination (MMSE) scores. - No effect on biomarkers of oxidative stress and inflammation.	Akbari et al., 2016
		Meta-analysis including 5 different studies (different probiotics).	- Improved cognitive performance by means of a decrease of inflammation and oxidative stress	Den et al., 2020
Depression	*Animal models*	Probiotic *Bifidobacterium infantis* chronically given to the offspring subjected early in life to the maternal separation paradigm model of depression.	- Normalization of the depressive-like behavioral deficits. - Restored basal noradrenaline concentrations and immune response.	Desbonnet et al., 2010
	*Clinical trials*	*Bifidobacterium longum* NCC3001 for 6 weeks	- Reduction in the depression scores. - Reduced limbic reactivity. - Increase in the quality of life.	Pinto-Sanchez et al., 2017
		Supplementation with the probiotics *Lactobacillus helveticus* and *Bifidobacterium longum*, or the prebiotic galacto-oligosaccharide for 8 weeks.	- Only the probiotic decreased in the Beck Depression Inventory (BDI) score.	Kazemi et al., 2019

### Neurodevelopmental Disorders

As far as studies on normal neurodevelopment are concerned, a two-center randomized placebo-controlled trial by Slykerman et al. (2018) aimed to determine whether probiotic supplementation early in life improves childhood cognition outcomes (intelligence, executive function, attention, depression, and anxiety) later in life (at 11 years of age). The authors investigated the effect of early probiotic by giving *Lactobacillus rhamnosus*, *Bifidobacteria animalis*, and *Bifidobacterium lactis* HN019 to mothers 35 weeks pregnant until 6 months and to their children from birth to 2 years. Overall, there were no significant differences in the neurocognitive outcomes (Slykerman et al., 2018). Even if it was unsuccessful, the main and most relevant finding of this study was that probiotics, even if given early in life during critical brain development, did not have a negative impact in normal brain function outcomes later in life.

Clinical studies exploited the use of probiotic in ameliorating the core symptoms of children with ASD. Two clinical trials reported improved GI symptoms (lower abdominal pain) by introducing prebiotics in the diet of children with ASD (Grimaldi et al., 2018; Sanctuary et al., 2019). Among the first clinical trials using probiotics, Kaluzna-Czaplinska and Blaszczyk (2012) investigated whether the probiotic *Lactobacillus acidophilus* could control the level of D-arabinitol (DA), a metabolite of most pathogenic *Candida* species found to be elevated in urine of autistic patients (despite the fact that there are data that endogenous DA might be derived also from the brain and not entirely attributable to *Candida*). The probiotic was given orally twice a day for 2 months, and it did interestingly decreased the DA level in the urine (Kaluzna-Czaplinska and Blaszczyk, 2012). In another study, Tomova et al. (2015) showed that a probiotic mixture of three strains of *Lactobacillus*, two strains of *Bifidobacteria*, and one strain of *Streptococcus* reduced levels of *Bifidobacteria* and *Lactobacillus* reduced levels of TNFα in the stools (Tomova et al., 2015). Shaaban et al. (2018) evaluated the efficacy and tolerability of probiotics *L. acidophilus*, *Lactobacillus rhamnosus*, and *Bifidobacteria longum* in an Egyptian cohort of children with ASD (trial number UMIN000026157). After 3 months of treatment, the stool from autistic children showed increases in the counts of *Bifidobacteria* and *Lactobacilli* levels, and a significant improvement in the severity of autism and GI symptoms, compared to the baseline (Shaaban et al., 2018). Santocchi et al. (2020) performed a double-blind randomized, placebo-controlled trial (NCT02708901) to determine the effects of supplementation with probiotics on specific GI symptoms, core ASD deficits, and cognitive and language development. The probiotic supplementation (De Simone Formulation) was a patented mixture (approved for use in children; marketed as Vivomixx^®^ in EU, Visbiome^®^ in United States) containing eight probiotic strains: *Streptococcus thermophilus*, *Bifidobacterium breve*, *Bifidobacterium longum*, *B. infantis*, *L. acidophilus*, *Lactobacillus plantarum*, *Lactobacillus para-casei*, and *Lactobacillus delbrueckii* subsp. *bulgaricus*. After 6 months of supplementation with the probiotics mixture, there was no effect when pooling all the data together, but differences were detected when grouping autistic children presenting or not presenting GI symptoms: in the group of children without GI symptoms, the authors reported a decrease in the severity of ASD score, while in the group with GI symptoms, the authors reported an improvement of the latter symptoms (Santocchi et al., 2020). Although the reviewed clinical trials were not standardized for the probiotics used (multiple different strains and concentrations were used) and varied for the duration of treatments, they opened up new and interesting non-invasive therapeutic avenues for ameliorating both the GI and the core symptoms in children with ASD.

As far as probiotic treatment in SSDs is concerned, given the increase in the abundance of gut *C. albicans* in male patients seen by Severance et al. (2016), the same group performed a randomized, double-blind, placebo-controlled trial (NCT01242371) over a 14-week period to test whether the manipulation of the intestinal microbiome by probiotics could ameliorate yeast imbalances, associated gut discomfort, and psychiatric symptoms. The probiotic formulation contained *Lactobacillus rhamnosus* strain GG and *Bifidobacterium animalis* subsp. *lactis* Bb12. The treatment reduced *C. albicans* antibodies in males, but not in females, and it showed a trend to improve the positive psychiatric symptoms (Severance et al., 2017) showing the effectiveness of probiotic treatment in restoring fungal gut imbalance and relieving SSD symptoms. Earlier, Dickerson et al. (2014) used the same probiotics formulation over a 14-week treatment trial finding no effect in reducing symptom severity, but the treatment did ameliorate bowel difficulty (Dickerson et al., 2014).

### Parkinson’s Disease

As discussed earlier, Parkinson’s disease is a cognitive and movement disorder characterized by the progressive degeneration of dopaminergic neurons in substantia nigra pars compacta resulting in dopamine deficiency in the striatum, and gut microbiota dysfunctions play a role in the pathogenesis of the disease, providing new targets for therapeutic interventions by means of the use of probiotics. Initial studies showed that (i) the use probiotics in the form of fermented milk containing *Lactobacillus casei shirota* improved stool consistency and defecation in people with Parkinson’s disease (Cassani et al., 2011), and that (ii) probiotics in tablets with *L. acidophilus* and *B. infantis* improved abdominal pain and bloating (Georgescu et al., 2016). The latter studies were followed by a randomized placebo-controlled trial showing that the intake of fermented milk containing fibers and different strains of probiotics relieved constipation in people with Parkinson’s disease (Barichella et al., 2016). Despite the fact that it is known that probiotics can increase gut motility (Waller et al., 2011) and constipation (Dimidi et al., 2017) in normal adults, it is currently unknown how they improve the GI symptoms in people with Parkinson’s disease. One randomized, double-blind, placebo-controlled clinical trial (IRCT2017082434497N4) (Tamtaji et al., 2019) assessed the impacts of probiotic supplementation on movement and metabolic parameters (assessed by means of The Movement Disorders Society-Unified Parkinson’s Disease Rating Scale, MDS-UPDRS) in individuals with Parkinson’s disease. The tested probiotics, capsules containing four strains (*L. acidophilus*, *L. reuteri*, *Bifidobacterium bifidum*, and *L. fermentum*), had useful impact on the MDS-UPDRS.

Infections caused by *H. pylori* decreased L-dopa absorption and increased motor function fluctuations in people with Parkinson’s disease (Pierantozzi et al., 2001). Since it has been shown that these effects are reversed by eradicating the pathogen (Pierantozzi et al., 2006; Hashim et al., 2014), two studies, one in wild type (normal) mice using probiotic supplementation of *B. bifidum* CECT 7366 (Chenoll et al., 2011) and one in humans using *L. reuteri* (Ojetti et al., 2012) (respectively), contribute to the eradication of the pathogen.

Since oxidative stress and peripheral immune activation do play a role in Parkinson pathogenesis, Magistrelli et al. (2019) used an *in vitro* approach to explore the effects of probiotic bacterial strains belonging to the *Lactobacillus* and *Bifidobacterium* genus on the peripheral blood mononuclear cells isolated from people with Parkinson’s disease and healthy controls, and found out that probiotics decreased pro-inflammatory cytokines and pathogenic bacterial overgrowth. First, the authors showed that all the strains used in the study did not carry the tyrosine decarboxylase genes, known to reduce the level of l-3,4-dihydroxyphenylalanine (levodopa) in people with Parkinson’s disease under treatment. Moreover, they found out that all probiotics tested reduced inflammatory cytokines and the production of reactive oxygen species (ROS) in both groups, with *L. salivarius* LS01 and *L. acidophilus* found to reduce pro-inflammatory and increase the anti-inflammatory cytokines with a higher extent in cells derived from people with Parkinson’s disease than from healthy controls (Magistrelli et al., 2019). Furthermore, most of the probiotics antagonized the growth of the pathogenic bacteria *E. coli* and *Klebsiella pneumoniae* (Magistrelli et al., 2019).

As far as probiotic effect on cognitive impairment is concerned, Xie and Prasad (2020) exploit the neurotoxin (6-hydroxydopamine, 6-OHDA) rat model of Parkinson’s disease to explore the impact of a probiotic treatment (*Lacticaseibacillus rhamnosus* HA-114) on anxiety and memory. After 6-OHDA-lesion of the striatum, (i) the authors assessed anxiety-like behavior using the elevated plus maze, and (ii) cognition was assessed for both non-hippocampal- and hippocampal-dependent memory using the novel object recognition and novel place recognition behavioral tests. Despite the fact that the probiotic *L. rhamnosus* had no impact on anxiety-like behavior induced by 6-OHDA-lesion, it did selectively improve hippocampal-dependent cognition deficits (Xie and Prasad, 2020) pointing to the potential of probiotics as adjunctive treatment for non-motor symptoms in Parkinson’s disease. However, while certain probiotic strains were shown to be effective for the treatment of cognitive symptoms of Alzheimer’s disease both in animal models (Bonfili et al., 2017, 2018) and in humans (Akbari et al., 2016), it is still unknown whether these therapies may be effective for alleviated cognitive symptoms also in people with Parkinson’s disease.

### Alzheimer’s Disease

As for Parkinson’s disease, the finding that the gut microbiota is altered in animal models and people with Alzheimer’s disease raises the possibility of the use of probiotics, which could be an effective therapeutic strategy for managing this neurodegenerative process and to ameliorate the cognitive pathological feature. Kobayashi et al. (2017) investigated the effects of oral subchronic (11 days) administration of *B. breve* strain A1 on the behavior and the physiological processes using a mouse model of the disease, namely, mice treated by intraventricular injection of Aβ. The authors found out that the treatment reversed the cognitive impairment assessed by the Y maze behavioral test and reduced the latency time in the passive avoidance test, thus indicating that the probiotic prevented the cognitive dysfunctions induced by Aβ. Moreover, the authors showed that *B. breve* strain A1 suppressed the Aβ-induced changes in gene expression of inflammation and immune-reactive genes in the hippocampus, and bacteria-derived metabolite acetate partially ameliorated the cognitive decline observed in the murine model (Kobayashi et al., 2017). In another work, Bonfili et al. (2017) assessed whether a modulation of the microbiota may induce positive effects on the neuronal pathways known to be able to slow down the progression of the pathology. In order to achieve this, the authors assessed whether treatment with the SLAB51 probiotic formulation (including lactic acid bacteria and bifidobacteria) could prevent the onset and affect the progression of the pathology in the triple-transgenic mouse model of Alzheimer’s disease, named 3xTg-AD. The SLAB51 probiotic formulation is known to affect the composition of gut microbiota and its metabolites by influencing plasma concentration of inflammatory cytokines and key metabolic hormones that were considered therapeutic targets in neurodegeneration. The mice that received the SLAB51 formulation showed (i) partial restoration of two impaired neuronal proteolytic pathways, namely, the ubiquitin proteasome system and autophagy; (ii) decreased cognitive decline, due to a reduction in brain damage; and (iii) reduced accumulation of Aβ aggregates (Bonfili et al., 2017). In a successive study, the same research group demonstrated that the SLAB51 formulation exerted a neuroprotective effect by reducing oxidative stress *via* the Sirtuin-1 (SIRT1) pathway, a NAD + -dependent protein deacetylase pathway (Bonfili et al., 2018). Thus, this research group demonstrated that a modulation of the microbiota by means of probiotics can slow down the progression of the disease.

As far as the effect of probiotics in people with Alzheimer’s disease is concerned, Akbari et al. (2016) performed a randomized, double-blind, and controlled clinical trial (IRCT201511305623N60) among 60 people with Alzheimer’s disease to assess the effects of a 12-week probiotic supplementation on cognitive function and metabolic status. The authors used a mixed-species product that included *L. acidophilus*, *L. casei*, *B. bifidum*, and *L. fermentum*. The oral chronic treatment improved the mini-mental state examination (MMSE) scores, while it had no effect on other biomarkers of oxidative stress and inflammation (Akbari et al., 2016). A recent meta-analysis including five studies involving 297 subjects indicated that probiotics improved cognitive performance in people with Alzheimer’s disease or mild cognitive impairment *via* decreasing inflammation and oxidative stress (Den et al., 2020).

Altogether, these studies in rodent models and humans clearly show the potential use of probiotic supplementation in preventing, ameliorating, and slowing down the cognitive deficits of Alzheimer’s disease.

### Depression

As far as depression is concerned, a study performed by Desbonnet et al. (2010) investigated the effect of the probiotic *B. infantis* in a very established model of depression, the maternal separation paradigm, where separation of the rat pups from the mother results in depression-like behaviors (Tractenberg et al., 2016). The authors applied the maternal separation protocol to the pups during the early postnatal life, and later in life, adults were chronically treated with either *Bifidobacteria* probiotic or citalopram (an commonly used antidepressant) and their depression-like behavior was assessed by means of the forced swim test (FST; a test centered on the rat response to the threat of drowning; rats with a depression-like behavior swim less than those not showing a depressive-like behavior). In control animals, as expected, maternal separation reduced swim behavior and increased immobility in the FST; moreover, it decreased noradrenaline (NA) content in the brain, and enhanced peripheral interleukin (IL)-6 release. Surprisingly, *Bifidobacteria* probiotic chronic treatment normalized the depressive-like behavioral deficits and restored basal NA concentrations and also the immune response, suggesting that probiotics (*Bifidobacteria* in this case) may have therapeutic applications in mood disorders (Desbonnet et al., 2010).

A randomized double-blind placebo-controlled clinical trial (NCT01276626) examined the effect of the *B. longum* NCC3001 in adults with IBS and also mild to moderate anxiety and/or depression (Pinto-Sanchez et al., 2017). The authors found out that after 6 weeks of probiotics treatment, there was a reduction in the depression scores (but not anxiety) together with an increase in the quality of life in comparison with the placebo. The improvement in depression was associated with changes in brain activation patterns assessed by means of functional magnetic resonance imaging (fMRI) that indicate that the probiotic reduces limbic reactivity. In another randomized double-blind placebo-controlled clinical trial, Kazemi et al. (2019) compared the effect of supplementation with the probiotics *Lactobacillus helveticus* and *B. longum*, or the prebiotic galacto-oligosaccharide on people with major depressive disorder. Eight weeks of probiotics supplementation resulted in a significant decrease in the Beck Depression Inventory (BDI) score in comparison, while the prebiotic supplementation had no effect in comparison to the placebo group (Kazemi et al., 2019). The reviewed studies performed in humans highlighted the potential therapeutic effect of probiotic in people with depression.

## Conclusion and Future Perspectives

In conclusion, the gut brain plays an important role in both the physiology and the pathology of the brain, as highlighted here in brain conditions spanning from neurodevelopmental disorders to Alzheimer’s and Parkinson’s diseases and depression. Even if their precise mechanism of action required further investigations, probiotic supplement treatments are valuable tools with beneficial future therapeutic avenues for neurological conditions. Many data recently emerged on dysbiotic states connected to neuropathological conditions, but still more clinical data are needed to demonstrate that correcting the dysbiotic state would restore physiological brain functions. Probiotics still come with limitations because the field is still poorly regulated by law and they are investigated in clinical trials as a “food supplement” rather than as a “drug.” Moreover, as shown in this review, there are many promising bacterial families that need to be addressed in clinical trials, but often they are not translated in probiotics simply because of the limited choice of bacteria available as probiotics. Probiotic supplementation occurs *via* oral intake, although few studies are available about the effective gut colonization of the bacterial strains present in probiotics. Nevertheless, different probiotic formulations guarantee microbe survival in their transit through the acidic pH of the stomach to reach the gut. In the near future, thanks to the continuing research on gut brain–brain interactions in physiological and pathological conditions together with the increment of available microbial strains suitable as probiotic, specific gut dysbiosis will be specifically tackled by targeted interventions in order to equilibrate/compensate the dysbiotic state.

## Author Contributions

Both authors conceptualized, wrote, read, and revised the manuscript.

## Conflict of Interest

MB is the CEO of BioArte. GD is a consultant for BioArte.

## Publisher’s Note

All claims expressed in this article are solely those of the authors and do not necessarily represent those of their affiliated organizations, or those of the publisher, the editors and the reviewers. Any product that may be evaluated in this article, or claim that may be made by its manufacturer, is not guaranteed or endorsed by the publisher.
